# Long sperm fertilize more eggs in a bird

**DOI:** 10.1098/rspb.2014.1897

**Published:** 2015-01-22

**Authors:** Clair Bennison, Nicola Hemmings, Jon Slate, Tim Birkhead

**Affiliations:** Department of Animal and Plant Sciences, University of Sheffield, Western Bank, Sheffield S10 2TN, UK

**Keywords:** sperm length, swimming velocity, fertilization success, sperm competition

## Abstract

Sperm competition, in which the ejaculates of multiple males compete to fertilize a female's ova, results in strong selection on sperm traits. Although sperm size and swimming velocity are known to independently affect fertilization success in certain species, exploring the relationship between sperm length, swimming velocity and fertilization success still remains a challenge. Here, we use the zebra finch (*Taeniopygia guttata*), where sperm size influences sperm swimming velocity, to determine the effect of sperm total length on fertilization success. Sperm competition experiments, in which pairs of males whose sperm differed only in length and swimming speed, revealed that males producing long sperm were more successful in terms of (i) the number of sperm reaching the ova and (ii) fertilizing those ova. Our results reveal that although sperm length is the main factor determining the outcome of sperm competition, complex interactions between male and female reproductive traits may also be important. The mechanisms underlying these interactions are poorly understood, but we suggest that differences in sperm storage and utilization by females may contribute to the outcome of sperm competition.

## 1. Introduction

Sperm competition is almost ubiquitous across the animal kingdom [1] and imposes strong selection on males to produce high-quality sperm. Males of species experiencing intense sperm competition typically produce ejaculates with: (i) more sperm [2], (ii) a higher proportion of viable sperm [3], (iii) more uniform sperm morphology [4–7], (iv) longer sperm [8–12], but see [13], and (v) faster swimming sperm [14,15], relative to males of species with little or no sperm competition.

Our understanding of how different sperm traits influence competitive fertilization success, however, remains incomplete. The number of sperm inseminated is often important in determining the outcome of sperm competition ([16–18] but see [19]), but the enormous variation in sperm morphology across species [20,21] suggests that size and shape are also important. However, attempts to understand how sperm length influences fertilization success have yielded inconsistent results (e.g. [19,22,23])—inconsistencies that may be partly explained through variable sperm competition mechanisms and ejaculate investment across different taxa [24].

Longer sperm are assumed to have an advantage over short sperm in a competitive scenario, because long sperm generally have: (i) longer flagella [10], providing greater forward propulsion [25], and (ii) relatively larger midpieces ([10], see also [26]), which produce more energy (via adenosine triphosphate (ATP) [27,28]. Although the relationship between sperm ATP content and swimming speed is uncertain [28–30], there is good evidence that longer sperm swim faster than shorter sperm, both within and between species (e.g. [15,31], but see [32,33]).

Faster swimming sperm are often assumed to fertilize more ova because fast sperm may reach the site of fertilization before slow sperm. This relationship between swimming speed and fertilization success is evident in some species of birds [34,35] and fish [19]. In species where longer or larger sperm achieve higher velocities, logic suggests that sperm size should then predict a given male's fertilization success in a sperm competition situation. In fact, there is limited experimental support for this prediction [36]. It is possible that, in some species, high levels of intra-ejaculate variation in male's sperm could mask a positive relationship between sperm length and fertilization success [31]. A lack of variation between sperm of an individual male (i.e. in species with intense sperm competition) could mean that detecting relationships is challenging (although not impossible, e.g. [37,38]).

In this study, we use the zebra finch, *Taeniopygia guttata*, to clarify the relationship between sperm length and fertilization success. The zebra finch is an ideal species to do this because considerable natural variation in sperm length exists between males (mean values for different males vary from approximately 40 to 80 μm [39], as a consequence of relatively low sperm competition intensity [39,40]. In previous studies of the zebra finch, we have also shown that: (i) sperm length is extremely consistent both within and between the ejaculates of individual males [41], (ii) length and swimming speed are heritable and positively genetically correlated [39,42], and (iii) longer sperm swim at greater velocities than shorter sperm [42]. Crucially, however, it is still not known whether, in a competitive scenario, males producing long sperm enjoy greater fertilization success than males producing relatively short sperm.

We conducted sperm competition experiments to test the hypothesis that, in a competitive environment, long sperm males fertilize more ova than short sperm males. In a mate-switching experimental design (similar to [43]), pairs of males, one male producing long sperm and the other producing short sperm, were mated sequentially, for 3 days per male, to a single female. In the zebra finch and other birds, inseminated sperm are stored in the female's reproductive tract in specialized sperm storage tubules (SSTs) [44–46] from which they are lost over time at a constant rate [47–49]. In birds, following sequential copulations with two different males, the proportion of sperm from the second mating male is expected to increase across successive eggs in a clutch. This is a result of passive sperm loss from the SSTs, such that fewer sperm from the first male remain in the SSTs at any given time point [50], explaining why, in birds, when all else is equal, sequential copulations usually result in the last male to copulate siring most offspring [43,51]. In our sperm competition experiments, we controlled for last male sperm precedence by employing a paired experimental design, in which we repeated the sperm competition protocol with the identical male pairs and females, but alternated the order in which the long and short sperm males copulated with the female.

By counting the sperm embedded in the outer perivitelline layer (OPVL) of the avian ovum, it is possible to estimate how many sperm reach the ovum and to determine the likelihood of fertilization following a single insemination [52]. We developed this technique further, using phenotypic ‘labelling’ of sperm, to allow us to confidently assign each individual sperm observed on the OPVL to one of the competing males. This allowed us to assess the proportion of each male's sperm that reached the ovum. We also determined the paternity of each embryo, revealing the eventual winner of sperm competition. In addition, we investigated whether an individual male's fertilization success in a sperm competition scenario is predicted by the number of his sperm reaching the ovum relative to that of the other male. Our results provide unique insight into the processes occurring immediately prior to fertilization, and how they affect the outcome of sperm competition.

## 2. Material and methods

### (a) Animals

The zebra finches in this study were part of a domesticated population maintained at the University of Sheffield since 1985. Zebra finch sperm morphology (e.g. sperm total length) is highly heritable [39,42]. We conducted an artificial breeding experiment (described in the electronic supplementary material) which increased the number of males in the population that produced long (more than 70 μm) or short (less than 60 μm) sperm, but did not increase sperm length beyond that which occurs naturally [39]. Sperm samples were collected from all adult male birds [53] and five morphologically normal sperm per male were photographed using light microscopy at 400× magnification (Infinity 3 camera, Luminera Corporation, and Leitz Laborlux microscope) and measured to the nearest 0.01 μm using ImageJ [54]. Based on these initial sperm measurements, pairs of males (matched by nearest hatching date) were selected for the sperm competition experiment, such that one male produced long sperm (*n* = 18) and one produced short sperm (*n* = 18). In no case did the sperm of the male pairs overlap in length (mean difference between males ± s.e.m.: 18.27 ± 0.70 μm). Each male pair was allocated to an unrelated (i.e. not a sibling, parent or offspring) female who originated from either the long (*n* = 8) or short (*n* = 10) selection line. The mean relatedness scores (presented as mean ± s.d.) between the male and female pairs, and between pairs of competing males were low (0.0371 ± 0.056 and 0.0026 ± 0.007, respectively; see the electronic supplementary material for further details). Females were housed singly in a cage (dimensions 0.6 × 0.5 × 0.4 m) with a nest-box half filled with hay. Each female cage had an adjoining cage for use later in the experiment.

### (b) Sperm competition experiments

Sperm competition experiments were conducted using a mate-switching protocol [43]. One male from each pair was paired to the female for 3 days (and allowed to copulate freely). The males were selected systematically to ensure that approximately half of the females (in both the long and short lines) were paired to a long sperm male first, with the remaining females paired to a short sperm male first. The second male (either a long or short sperm male) was then paired to the female for an additional 3 days (to copulate freely). After 3 days, the second male was placed in the adjoining cage, where a wire mesh divider prevented any further physical contact. Females were allowed to lay a clutch of eggs, all of which were collected daily (*n* = 192) and marked with a unique female code and the egg number. Eggs were artificially incubated at 38°C for 48 h, and stored at 4°C until processing. When the duration of sperm storage for female zebra finches was exceeded—14 days [55]—each mating trial was repeated as above (using the identical males and females), except males were paired to the female in the reverse order. Thirty clutches of eggs were collected and analysed from 18 females; 12 of which produced a clutch of eggs in both mating rounds.

### (c) Quantifying competitive success

Male competitive success was assessed in two ways: (i) the proportion of sperm from each male that reached each ovum (determined by counting sperm on the OPVL) [47] and (ii) the paternity of each embryo. Eggs were dissected in the following way, as in [56]. The egg was opened into a petri dish of phosphate-buffered saline (PBS), and the embryo gently detached from the surface of the yolk using a hair loop (a piece of human hair taped to a pipette tip to form an oval loop approx. 5 mm long), collected using a pipette and sterile pipette tip, and stored in 100% ethanol for molecular paternity analysis at a later date. The yolk was cut in half and the OPVL was removed, washed in PBS, laid flat on a microscope slide, stained with 10 μl Hoescht 33342 fluorescent dye (0.5 mg ml^−1^) (Molecular Probes, USA) and incubated in the dark for 2 min. We examined the half of the OPVL that contained the germinal disc (GD) because the majority of sperm are observed around the GD [57]. Using fluorescence combined with darkfield microscopy (Leica DMBL) at 400× magnification, sperm on the OPVL were photographed (Infinity 3 camera, Luminera Corporation), and sperm length (*n* = 4420) was measured to the nearest 0.01 μm [54] (see the electronic supplementary material for images of sperm embedded in the OPVL). This measurement was used to assign each sperm to either the long or short sperm male based on sperm length data collected previously; thus, each male's sperm in the OPVL was ‘labelled’ by its phenotype (long or short). The mean length of sperm collected directly from the male (from the seminal glomera—SG—see below), and from sperm embedded in the OPVL (from the same male), was significantly correlated (*r*^2^ = 0.96, *t* = 15.18, d.f. = 18, *p* < 0.0001). In cases where the sperm's head was missing, we used flagellum length to identify sperm as long or short (flagellum and total length are also significantly correlated; *r*^2^ = 0.99, *t* = 106.01, d.f. = 33, *p* < 0.0001).

### (d) Sperm quality analyses

At the end of the experiment, all males (fully rested from copulation for at least four weeks) were humanely killed by cervical dislocation and sperm collected from the distal region of the left SG by dissection. The following sperm quality analyses (described in the electronic supplementary material) were carried out to determine whether sperm quality parameters were similar within the male pairs: (i) swimming velocity (the swimming speed of sperm), (ii) viability (the proportion of viable sperm), (iii) morphology (the proportion of sperm with normal, undamaged morphology), (iv) concentration, and (v) longevity (the length of time sperm remained motile) (for results, see the electronic supplementary material, table S1). Testes mass data were also collected (electronic supplementary material, table S2). Data on copulation rate and SG mass were opportunistically collected from long and short sperm males that were not used in the experiment (refer to the electronic supplementary material, tables S3 and S4).

### (e) Paternity assignment

DNA was extracted from embryos using the ammonium acetate protocol [58]. DNA was amplified by PCR using a DNA Engine Tetrad 2 thermocycler (MJ Research, Bio-Rad, Hemel Hampstead, Herts, UK). The PCR products were genotyped using an ABI 3730 48-well capillary sequencer (Applied Biosystems, CA, USA). The reaction products were visualized and scored for eight microsatellite loci using GeneMapper v. 3.7 (Applied Biosystems, CA, USA). Paternity was assigned to embryos (*n* = 166) using Cervus v. 3.0.3 [59], at greater than 80% confidence. For detailed methods, see the electronic supplementary material.

### (f) Data analysis

All data were analysed in R v. 2.15.1 [60]. Exact binomial tests were used to test for differences in the numbers of long and short sperm that reached the OPVL, and the number of embryos sired by the long and short sperm males. Generalized linear mixed models (GLMMs) in the R package LME4 [61] were used to investigate whether male sperm length determined fertilization success. Data were modelled using the function ‘glmer’ with a binomial error distribution and logit link function. To determine the relationship between the proportions of long sperm reaching the ovum and the likelihood of the long male siring the embryo, we first modelled embryo paternity as either ‘1’ or ‘0’ (i.e. sired by the long male or not), with the proportion of long sperm embedded on the OPVL included as a fixed effect. Trio ID (i.e. a single female and pair of males) was used as a random effect.

In order to control for the effects of last male sperm precedence (we repeated the experiment with males copulating in the reverse order), we then carried out a second GLMM that used the second mating male as the focal male in the analysis. The paternity of each embryo was included as either ‘1’ or ‘0’ (i.e. sired by second male or not). Male mating order (short first/short second), female line (long/short) and the number of days between the male swap and the laying of the focal egg were included as fixed effects. Trio ID was fitted as a random effect. We also modelled all interactions between the three fixed effects. Model simplification was carried out using log-likelihood tests and Akaike information criterion (AIC) values to obtain the minimal adequate models.

## 3. Results

### (a) Sperm length influences fertilization success

**Figure 1.**
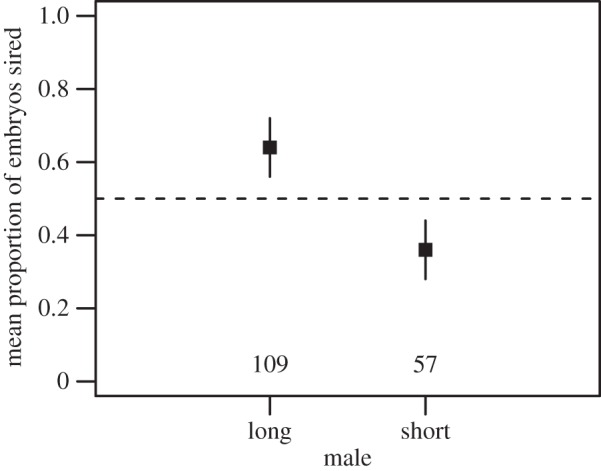
Long sperm males sired a greater proportion of embryos compared with the short sperm males (see base of plot for numbers of embryos sired by the long sperm male in each group). *N*_total_ = 166. Bars represent standard errors and the dashed line at *y* = 0.5 represents the expected proportions if sperm length did not influence fertilization success. See main text for further description of the data.

**Figure 2.**
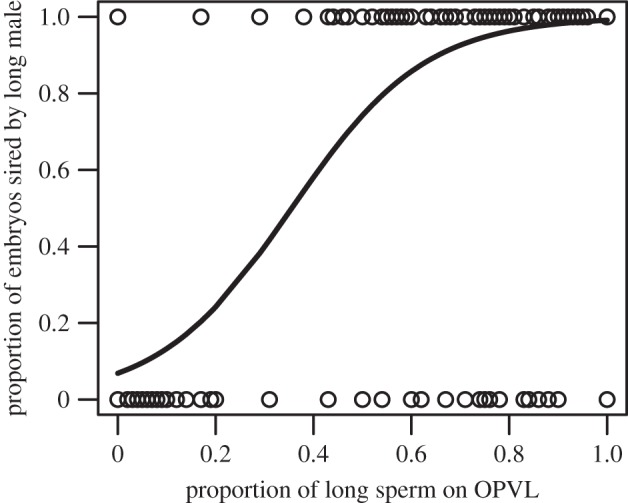
The proportion of embryos sired by the long sperm male increases as more long sperm are observed on the OPVL. Data comprise 192 eggs, of which paternity was assigned to 166 embryos. The line is the fitted logistic model.

Significantly more long sperm (57 ± 2%) reached the ova than short sperm (43 ± 2%) (mean percentage ± s.e.m. of sperm counts; exact binomial test; *p* < 0.0001). Long sperm males sired a greater proportion of embryos (64 ± 8%) than short sperm males (36 ± 8% (mean percentage ± s.e.m. of all paternity results; exact binomial test; *p* < 0.0001; figure 1; see also the electronic supplementary material, table S5). Sperm total length and swimming velocity differed between the competing males (electronic supplementary material, table S1), such that longer sperm swam faster, as in [42]. Our results also show that the proportion of sperm on the OPVL from a given male determines his likelihood of successful fertilization (GLMM; estimate = 7.86 ± 1.42 (mean ± s.e.m.); *z* = 5.52; *p* < 0.0001; figure 2).

### (b) A lack of last male sperm precedence

Mating order of the males did not determine which male fertilized the egg (long male first: 69 ± 10% (mean percentage ± s.e.m.); long male second: 60 ± 11% (mean percentage ± s.e.m.); proportion test; *χ*^2^ = 1.15, *p* = 0.28)). This means that the patterns of paternity observed in this study cannot be explained simply by the passive loss of sperm from the SSTs, where last male precedence would be expected [50].

### (c) Male × female interaction

**Figure 3.**
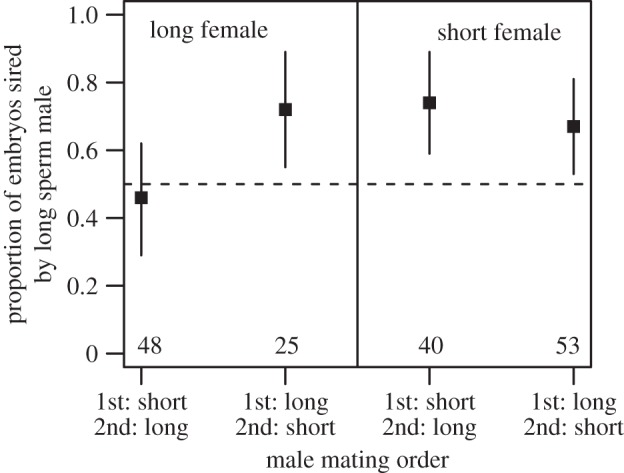
The proportions of embryos sired by the long sperm male according to male mating order and the selection line of the female (long or short). The long sperm male fertilized more ova in three out of the four combinations of male mating order and female line (see base of plot for numbers of embryos sired by the long sperm male in each group). *N*_total_ = 166. Bars represent standard errors and the dashed line at *y* = 0.5 represents the expected proportions if sperm length did not influence fertilization success. See main text for further description of the data.

Male fertilization success was also influenced by interacting effects of male mating order and female selection line (GLMM; estimate = 3.60 ± 1.12; *z* = 3.20, *p* = 0.001, figure 3; see the electronic supplementary material, table S6, for model output). The number of days between the male swap and the laying of the focal egg did not affect male fertilization success as a main effect, nor did it interact significantly with any other factors. Long sperm males sired more embryos than short sperm males in three out of the four mating combinations: (i) long male first, short male last, long female, (ii) long male first, short male last, short female, and (iii) short male first, long male last, short female. However, in a single mating combination (short male first, long male last, long female), the proportion of embryos sired by the long and short sperm males were not significantly different from 0.5 (exact binomial test; *p* = 0.89; see the electronic supplementary material, table S5, for summary data). Taken together, these results demonstrate that in the zebra finch, long sperm males are more successful in a sperm competition scenario than short sperm males.

## 4. Discussion

We have experimentally demonstrated for the first time, we believe, in a vertebrate species that under competitive conditions, long sperm tend to reach ova in greater numbers, and consequently fertilize a greater number of ova than short sperm. Our controlled experimental design, which incorporated a powerful pairwise comparison of male fertilization success using alternate mating of males, revealed an apparent lack of last male sperm precedence. This is inconsistent with the passive sperm loss model of last male sperm precedence, which is the widely accepted mechanism of sperm competition in the zebra finch [50] and other birds (e.g. [62]). The passive sperm loss model predicts that all else being equal (including sperm length and swimming velocity), following sequential inseminations by two different males, a greater proportion of eggs should be fertilized by the second male to copulate. However, in this study, we found that regardless of whether they were first or second to copulate, long sperm males sired significantly more embryos than short sperm males in the majority of pair combinations. Surprisingly, the only scenario in which this was not the case was when the long sperm males copulated second (and were therefore predicted—because of last male sperm precedence—to have had an advantage regardless of sperm length) with females who originated from the long sperm selection line. In this particular instance, the proportion of embryos sired by males from both lines did not differ significantly from 0.5, so it is difficult to draw any conclusions about this particular result in isolation.

The simplest explanation for the observed overall long sperm advantage would be that, because long sperm swim faster (electronic supplementary material, table S1, and [42]), they reach the SSTs sooner than short sperm. However, this is unlikely to account for the patterns of paternity we observed for the following reason. Assuming that space in the SSTs is limited, and long sperm reach the SSTs sooner, the ‘fertilizing set’ of sperm in the SSTs would therefore consist of a higher proportion of long sperm than short sperm. As a result, more long sperm would reach the ovum, increasing the odds of a long sperm fertilizing the ovum. This result is what we would expect if the two inseminations (of long and short sperm) occurred simultaneously—effectively as a single, mixed insemination (as in [51]). In our experiment, however, inseminations were sequential, with the first male copulating with the female for 3 days, after which he was replaced with the second male who also copulated for 3 days. Despite this interval between inseminations, mating order did not affect the outcome of sperm competition, because the long sperm males generally sired the majority of embryos. This indicates that there may be differences in the rates of uptake or release of long and short sperm into or from the SSTs, which may influence the relative proportions of long and short sperm available at the time of fertilization.

In a study of domestic fowl (*Gallus gallus domesticus*) [63], it was found that high mobility sperm (when mobility is measured as the ability of sperm to penetrate a solution of inert medium (Accudenz), which is positively correlated with sperm swimming velocity [64]) fertilized more ova overall than low mobility sperm under sperm competition, and that this relative success increased over successive eggs within the clutch. One explanation for these results is that high mobility sperm remain in storage for longer than low mobility sperm, which is consistent with an earlier hypothesis [65]. In this study, since long zebra finch sperm swim faster than short sperm (electronic supplementary material, table S1), this may also explain why long sperm males achieved higher paternity regardless of mating order.

Alternatively, our results may be accounted for if short sperm are simply less likely to reach and/or enter the SSTs. To reach the uterovaginal junction, where the SSTs are located, sperm must swim through the hostile vaginal region of the oviduct, so it is likely that swimming speed determines success during this phase [66]. Again, since we know that short sperm swim more slowly than long sperm, it is possible that fewer short sperm than long sperm (in absolute terms) are able to survive the journey through the vagina to the SSTs. This could result in a greater proportion of long sperm in the ‘fertilizing set’, regardless of mating order. This is a particularly interesting idea, given that our results suggest that the long sperm males may store fewer sperm (although not significantly fewer) prior to copulation (electronic supplementary material, table S1). If we speculate that the stored sperm concentration may be related to the number of sperm used for insemination (note that we could not test this relationship), this suggests that the long sperm fertilization advantage reported in this study may be a conservative estimate.

Overall, long sperm outcompeted short sperm in our study, but sperm length was not the only factor influencing fertilization success. Specifically, the selection line origin of the female also appeared to influence the degree of last male precedence in our sperm competition trials. Assuming an overriding long sperm advantage, as our results indicate, data from matings with females from the short selection line also suggest a small underlying effect of last male precedence. As expected, long sperm males are more successful in both cases, but less so when the short sperm male was second to mate; figure 3). Data from matings with long line females, however, suggest the opposite pattern—an unexpected underlying effect of *first* male precedence (figure 3). Without further experiments, it is difficult to explain these opposing patterns across female lines, but this result is suggestive of a female-mediated influence on the outcome of sperm competition.

There is increasing evidence that females exert some control over paternity [67], and that the final outcome of sperm competition may be determined by a combination of both male and female effects [68–71]. In *Drosophilia*, for example, sperm are stored in the female's seminal receptacle (SR), and the size and shape of her SR influences a male's fertilization success depending on his sperm length. In an elegant experiment, Miller & Pitnick [70] used populations of male and female *Drosophila*, artificially selected for divergence in sperm length and SR length, respectively. Long sperm males had a pronounced fertilization advantage when copulating with females with long SRs, possibly due to optimal positioning of long sperm within the SR for fertilization. Given the growing evidence of the pivotal roles of females in determining the outcome of sperm competition, particularly in internally fertilizing species, it is perhaps unsurprising that, in addition to the strong effect of sperm length, we also found some evidence for female effects on competitive fertilization success in the zebra finch.

## 5. Conclusion

We have experimentally demonstrated that in the zebra finch, long sperm have an advantage in sperm competition compared with short sperm. This long sperm advantage is evident both in the number of sperm that reach the site of fertilization and those that fertilize the ovum. As all other measures of sperm quality, except swimming velocity, were comparable between our long and short sperm males, the competitive success of the long sperm males can clearly be attributed to sperm length. Importantly, however, our results demonstrate that male competitive success is not necessarily the simple outcome of a race between the sperm of rival males. Instead, sperm competitive success appears to be mediated by the female, possibly through as yet unknown mechanisms of differential sperm acceptance or release from sperm storage sites.

## Supplementary Material

Selective breeding methods

## Supplementary Material

Long and short sperm male comparisons

## Supplementary Material

Quantifying competitive success

## Supplementary Material

Relatedness between experimental birds

## Supplementary Material

Sperm competition experiment summary tables

## Data accessibility

The following datasets relevant to this study are available from the Dryad digital repository (http://www.datadryad.org; doi:10.5061/dryad.dc335): (i) sperm competition experimental data, (ii) sperm quality comparisons, (iii) testes mass data, (iv) seminal glomera mass data, (v) copulation rate data, and (vi) relatedness scores between experimental birds.
