# Supplementary material for: Long sperm fertilize more eggs in a bird
Source: Proc Biol Sci. 2015 Jan 22;282(1799):20141897. doi: 10.1098/rspb.2014.1897 (PMC4286041; doi:10.1098/rspb.2014.1897)
Supplement: Selective breeding methods [file rspb20141897supp1.docx]

Selective breeding methods

All zebra finches used in this study were from a domesticated, outbred, pedigreed population that has been maintained at the University of Sheffield since 1985. The birds were housed at 18-24^o^C on 14:10 light: dark cycle using Osram L 36W/965 Deluxe Cool Daylight light bulbs controlled by a Lutron Grafik integrale GXI-3000 system. The birds received a standard zebra finch diet with ratios of 4:4:2:2:2:1 of white, panicum and yellow millet, canary seed, Japanese and red millet (Haiths, UK). Water, grit and cuttlefish were available ad libitum, supplemented weekly by lettuce, orange, millet spray and Abidec^®^ liquid vitamins (approximately 3 drops per litre of water). Breeding pairs with chicks received egg-food (soaked seed and boiled egg) daily.

Three selection lines were created in October 2009 from the population of zebra finches: long (L-line), intermediate (I-line) and short (S-line) to increase the numbers of males producing extremely long or short sperm. Three cohorts of zebra finches were produced over three years (2009 - 2011). The estimated breeding values (EBVs) for all zebra finches were computed using the Animal model [1] in AsREML v2.0 (in 2009) [2] and MCMCglmm (in 2010-11) [3], where positive and negative values infer long and short sperm phenotypes, respectively. Both methods used an extensive pedigree of the whole population (n > 8000 birds) and the sperm total length of each male (see below). Although females do not produce sperm, the Animal model computes EBVs for sperm length for each female using the phenotypic data from her male relatives. Thus, females that are expected to produce sons with long or short sperm can be identified.

The EBVs were imported into a custom-made database for the zebra finch long-term study (Sunadal Data Solutions), and a list of male - female pair combinations (male and female EBVs within ± 1.0 unit) and the pair-average EBVs were obtained. Pair-average EBVs were sorted numerically and pairs of birds with the most extreme positive and negative average EBVs were selected for the L- and S-line respectively (30 for each selection line). Thirty pairs with a pair average EBV approximately zero were selected for the I-line. The database ensures that each male and female in a pair are unrelated, such that they do not share grandparents.

Each pair was housed in a cage (dimensions 0.6 x 0.5 x 0.4m) with a nest box half filled with sterile hay and boxes were checked daily. Egg lay date and hatch dates of chicks were recorded. Chicks were weighed on the morning of hatching (to the nearest 0.01 g) using a Sartorius Acculab balance, and marked using permanent non-toxic marker pens for identification. Weights were recorded on 5, 10, 20, 30 and 40 d post hatching. Chicks were ringed with a closed metal identification ring on the right leg at 10 d old. Juveniles were separated from the parents when the youngest chick reached 40 d old and housed in mixed sex groups of multiple families until sexual maturity at around 100 d.

A sperm sample was collected from each male zebra finch at 100 d. Five sperm with a normal morphology (i.e. undamaged and no developmental abnormalities) were measured to give the phenotype of each male [4]. Sperm measurement data were used to re-calculate new EBVs prior to selecting the pairs for the next breeding cohort for each selection line.

References

1. Kruuk LEB. 2004 Estimating genetic parameters in natural populations using the 'animal model'. *Philos. Trans. R. Soc. Lond. Ser. B-Biol. Sci.* **359**, 873-890. (doi:10.1098/rstb.2003.1437).

2. Gilmour AR, Gogel BJ, Cullis BR & Thompson R. 2006 ASReml User Guide (2.0 ed, VSN International Ltd, Hemel Hempstead, HP1 1ES, UK.

3. Hadfield JD. 2010 MCMC methods for multi-response Generalized Linear Mixed Models: The MCMCglmm R Package. URL http://www.jstatsoft.org/v33/i02/. *J. Stat. Softw.* **33**, 1-22.

4. Birkhead TR, Pellatt EJ, Brekke P, Yeates R & Castillo-Juarez H. 2005 Genetic effects on sperm design in the zebra finch. *Nature* **434**, 383-387. (doi:10.1038/nature03374).
