# Supplementary material for: Long sperm fertilize more eggs in a bird
Source: Proc Biol Sci. 2015 Jan 22;282(1799):20141897. doi: 10.1098/rspb.2014.1897 (PMC4286041; doi:10.1098/rspb.2014.1897)
Supplement: Long and short sperm male comparisons [file rspb20141897supp2.docx]

Comparisons between long and short sperm males

Sperm quality assays

Sperm for the following assays were obtained by dissection of the left seminal glomerus (SG) of all male zebra finches used in the sperm competition trial (n_total_ = 36; n_long_ = 18; n_short_ = 18). A small amount of concentrated sperm (approximately 1 mm length of the SG) were squeezed from the distal region of the SG (nearest to the cloaca) into warmed nutrient media (Ham’s F10: Invitrogen, UK). Sperm solution (concentration approximately 8 x10^6^ per ml) was collected using a pipette after allowing sperm to ‘swim out’ in the media for 10 s.

a) Sperm velocity

Sperm velocity was analysed using the Sperm Class Analyzer® (Microptic, Barcelona, Spain). Sperm videos were captured using pseudo negative phase at 200x magnification with a Basler acA780-75gc camera connected to an Olympus BX41 microscope. Four microlitres of sperm solution was loaded into a 20 µm depth slide chamber (Leja®, Netherlands) and allowed to equilibrate on the microscope heated stage (heated to 38^o^C) for 30 s. Multiple 1 s video clips per male were recorded in a systematic manner until at least 100 sperm had been tracked.

Debris was manually deleted from all videos prior to analysis. Three kinematic parameters from each sperm were obtained: (i) average path velocity (VAP), (ii) curvilinear velocity (VCL), and (iii) straight line velocity (VSL). The kinematic values of drifting sperm were set to zero (i.e. non-motile) when the following conditions were met: VAP < 7.5 µm/s; VCL < 14 µm/s; and VSL < 2.5 µm/s. These values were obtained by analysing videos of dead sperm drifting, using the same protocol as above. Due to co-linearity of VAP, VCL and VSL, a principle components analysis was used to obtain a single index of sperm swimming velocity (PC1) for each male.

b) Sperm morphology, and percentage of sperm with normal morphology

Five microlitres of sperm solution was fixed in 5% formalin. Ten morphologically normal, undamaged sperm were photographed using light microscopy (Leitz Laborlux S) at 400x and an Infinity 3 camera (Luminera Corporation) for males involved in the experiment. Although measuring only five sperm captures the majority of the morphological variation in a male’s sperm sample [1], measuring ten sperm ensured that we obtained virtually all sperm morphological variation. This was crucial because this sperm length data set provided the range of lengths of each sperm component (e.g. midpiece, total length) that were used to distinguish between long and short sperm on the outer perivitelline layer of eggs. The length of all sperm components was measured directly from the photographs (i.e. ‘simple’ midpiece length etc.) to the nearest 0.01 µm using ImageJ [2]. Observer measurement repeatability [3] was high for all sperm components (head: F = 74.9: r = 0.97; midpiece: F = 1390.1: r = 0.99; tail: F = 616.3: r = 0.98). Two hundred randomly chosen sperm were then scored as having normal or abnormal morphology, and the proportion of sperm with a normal morphology (no damage to any component part or visible morphological abnormalities) was calculated.

c) Sperm viability

Sperm viability was estimated using a membrane integrity assay (Live/Dead Viability kit (Invitrogen™)), prepared as follows: 1 µl of 2.4 mM propidium iodide (PI) was diluted using 200 µl PBS. One microlitre of 1 mM SYBR14 was diluted with 10 µl of dimethyl sulphoxide (DMSO). A small sample (0.5 µl) of sperm solution was collected and placed on a microscope slide. Three microlitres each of PI and SYBR14 were incubated with the sperm sample in the dark for 5 minutes. Using a fluorescent microscope (Leica DMBL) and 200x magnification, a minimum of 200 sperm were scored as having an ‘intact’ membrane or an ‘damaged’ membrane according to the colour of the nucleus (green = intact membrane, red = damaged membrane). The proportion of sperm with an intact membrane was calculated.

d) Sperm longevity and sperm concentration

The remaining sperm in the distal region of the left SG were carefully squeezed into 15 µl of media (at room temperature), a process that took approximately 5 minutes per bird. The concentrated sperm solution was divided into 1 µl aliquots, diluted with 3 µl of media and incubated at 38^o^C in a water bath. Every 15 minutes, an aliquot was diluted with warm media (38^o^C) to a concentration of approximately 8x10^6^ sperm per ml and loaded into a slide chamber. Five video clips per time point were recorded and the average proportion of motile sperm calculated. The duration of motility (in minutes) of each sperm sample was recorded at the time that all sperm were immotile.

A single aliquot was used to calculate the concentration of sperm in the distal region of the SG. Ninety-nine microlitres of 5% formalin was added to the sample before vortexing for 20 s (Autovortex mixer SA2) to disperse sperm clumps. Twenty microlitres of the sample was loaded onto an Improved Neubauer chamber. All sperm were counted across the both grids and an average of the two grids was calculated, giving the number of sperm found in a volume of 0.9 mm^3^. The sperm concentration per millilitre (ml), corrected for dilution, was calculated using the following formula:

$\text{Sperm concentration}\cdot{10}^{6}(\text{ml})=\left( \left( \frac{\text{sperm count}}{0.9} \right)\cdot1000 \right)\cdot100$

Sperm quality between the specific pairs of males used in the sperm competition experiments (one long sperm and one short sperm male) were analysed in R v 2.15.1 [4], using paired t tests, to establish whether there was any systematic bias in any measure of sperm quality that might influence the outcome of sperm competition.

The sperm quality of the long and short sperm males was similar in terms of the proportions of viable, morphologically normal and motile sperm (table S1). The distal region of the SG of short sperm males contained more sperm compared to the long sperm males, but this trend was non-significant. Long and short sperm males differed in sperm length (e.g. head, tail and total length), and sperm swimming velocity, such that long sperm swam faster than short sperm. Interestingly, mean midpiece length was similar between the long and short sperm male pairs, although there was less variation in the midpiece length of short sperm compared to long sperm.

Testes mass

The left testes of males were removed by dissection, cleaned with phosphate buffered saline (PBS), blotted dry and weighed to the nearest 0.001 g using a Sartorius Acculab balance. The effect of male selection line on left testes mass was analysed using a linear model in the base package in R [4], with male selection line (long or short) as a fixed effect. Body mass was included as a covariate. Left testes mass only was used in the analysis because sperm from the left SG only was collected for the sperm quality analyses.

Short sperm males had a lower left testes mass compared to long sperm males (table S2); on average, the testes of the short sperm males were 0.005 g lighter (18%). It is not possible to extrapolate the total number of sperm available for insemination from the mass of a single testis. However, if smaller testes produce fewer sperm, this could suggest that males with smaller testes may be disadvantaged in a competitive scenario. Interestingly, in this study, the short sperm males tended to store more sperm (although not significantly more) in the distal region of the left SG than long sperm males (table S1). At the very least, this data indicates that the lighter testes of the short sperm males were not a disadvantage during sperm competition.

Table S1. Results of the sperm quality comparisons between pairs of long and short sperm males used in the sperm competition experiments. Only measures of sperm length (with the exception of midpiece length) and swimming velocity differed within the pairs of males.

|  | **Mean ± S.D** | | **Test statistic** | | |
| --- | --- | --- | --- | --- | --- |
| **Sperm quality assay** | **Long** | **Short** | **t** | **p** | **n** |
| Proportion of motile sperm^1^ | 0.87 ± 0.12 | 0.88 ± 0.14 | 0.03 | 0.98 | 17 |
| Proportion of normal sperm | 0.74 ± 0.08 | 0.72 ± 0.14 | 0.21 | 0.84 | 17 |
| Proportion of viable sperm | 0.74 ± 0.11 | 0.75 ± 0.14 | -0.72 | 0.48 | 17 |
| Sperm concentration (x10^6^ per ml) | 107.70 ± 95.27 | 142.02 ± 117.60 | -1.22 | 0.24 | 17 |
| Sperm longevity *Proportion motile at t_0_^2^* | 0.63 ± 0.24 | 0.60 ± 0.29 | 0.60 | 0.55 | 17 |
| *All immotile (min)* | 113.33 ± 50.47 | 135.5 ± 75.75 | -1.35 | 0.19 | 17 |
| Sperm velocity (PC1) *Total population* | 1.51 ± 0.79 | 0.50 ± 0.71 | 6.32 | **<0.0001** | **17** |
| *Fastest 10%* | 3.62 ± 0.76 | 2.10 ± 0.72 | 10.39 | **<0.0001** | **17** |
| *Fastest single sperm* | 4.27 ± 0.75 | 2.95 ± 0.97 | 5.00 | **0.0001** | 17 |
| Sperm morphology (µm) *Head* | 11.42 ± 0.58 | 10.72 ± 0.43 | 4.34 | **0.0005** | **17** |
| *Midpiece* | 29.75 ± 5.37 | 31.06 ± 1.66 | -1.20 | 0.25 | 17 |
| *Tail* | 33.56 ± 6.42 | 14.77 ± 2.43 | 10.97 | **<0.0001** | **17** |
| *Total length* | 74.73 ± 2.19 | 56.55 ± 1.58 | 25.98 | **<0.0001** | **17** |

Comparisons between the male pairs were made using paired t tests to establish if there was systematic bias in sperm quality that could affect the outcome of sperm competition. Proportion data were arcsine transformed before analyses. Significant values are in bold (p < 0.05). N = number of paired comparisons. Note that 17 pair comparisons were carried out because sperm could not be obtained from one of the males.

^1^Calculated from data obtained during the velocity assay

^2^t_0_ denotes the proportion of motile sperm at the start of the longevity assay.

Table S2. The results of the linear model analysing the effect of male selection line on left testes mass. Males from the short selection line had lighter testes compared to long line males.

|  | Estimate ± SEM | t | p |
| --- | --- | --- | --- |
| Intercept | 0.0153 ± 0.0095 | 1.62 | 0.114 |
| Selection line: short | -0.0054 ± 0.0024 | -2.21 | **0.034** |
| Body mass (g) | 0.0008 ± 0.0005 | 1.48 | 0.148 |

Data comprise the combined left testes mass from all males used in the sperm competition experiment (n_long_ = 18; n_short_ =18).

References

1. Bennison C. 2014. Sperm morphology and fertilisation success in the zebra finch *Taeniopygia guttata.* PhD Thesis, The University of Sheffield*,* UK. 182-182.

2. Schneider CA, Rasband WS & Eliceiri KW. 2012 NIH Image to ImageJ: 25 years of image analysis. *Nat. Methods* **9**, 671-675. (doi:10.1038/nmeth.2089).

3. Lessells CM & Boag PT. 1987 Unrepeatable repeatabilities - a common mistake. *Auk* **104**, 116-121.

4. R Development Core Team. 2012 R: A language and environment for statistical computing. R Foundation for Statistical Computing, Vienna, Austria. See: <http://www.R-project.org/>.

Copulation rate of long and short sperm males

Using long and short sperm males that were not included in the sperm competition experiment, additional data were collected to exclude additional factors that could result in a competitive advantage of the long sperm males.

The copulation rate of long and short sperm males was obtained by analysing videos recordings of within pair copulation behaviour from initial introduction (between 14:00-15:00 h) and daily (07:00-12:00 h) until clutch initiation. Females from both the long and short selection lines were used (n = 7 from each line). Eleven females were trialled with both long and short sperm males. The number of copulation attempts was counted on the 5 days prior to clutch initiation [1]. All videos were analysed blind with respect to male and female identity and line. Copulation rate was analysed using a Generalised linear mixed model (GLMM) with a Poisson error distribution and a log-link function in the R package LME4 [2]. Male and female selection line, the number of days between pairing and clutch initiation, and the interaction between them were included as fixed effects. Male and female identities were random effects.

There was no difference in the rate at which long and short sperm males copulated with females, suggesting that the amount of sperm transferred to females may be similar (table S3). Copulation rate was also unaffected by female selection line (table S3). There was no interaction between male and female line on male copulation rate; therefore, the interaction term was removed from the final model.

Table S3. The result of the GLMM analysing the effect of male and female selection line identity on copulation rate. Male and female selection line did not influence the copulation rate of males.

|  | Estimate  ± SEM | z | p |
| --- | --- | --- | --- |
| Intercept | -0.018 ± 0.266 | -0.069 | 0.945 |
| Male line | 0.068 ± 0.257 | -0.265 | 0.791 |
| Female line | 0.051 ± 0.180 | 0.282 | 0.778 |
| Number days until clutch initiation | 0.091 ± 0.055 | 1.640 | 0.101 |

Data comprise 123 copulations from 16 males when paired to 14 females. Eleven females were paired to both a long and short sperm male.

References

1. Birkhead TR, Hunter FM & Pellatt JE. 1989 Sperm competition in the zebra finch, *Taeniopygia guttata*. *Anim. Behav.* **38**, 935-950.

2. Bates DM, Maechler M & Bolker BM. 2012 lme4: Linear mixed-effects models using S4 classes. R package version 0.999999-0. See http://CRAN.R- project.org/package=lme4.

Seminal glomera mass of long and short sperm males

Sperm are produced in the testes and are transported down the vas deferens, where they are stored in SG prior to ejaculation. The SG is the coiled distal region of the vas deferens (adjacent to the cloaca), and the amount of sperm stored in the SG can be inferred from the SG mass [1]. We used SG mass to establish whether the amount of sperm available for copulations differed between long and short sperm males

SGs from both left and right body sides were collected opportunistically from long (n = 6) and short line males (n = 6) that were not part of the sperm competition experiment. SGs were removed by dissection, stripped of fat and excess tissue, and washed in phosphate buffered saline (PBS) before weighing to the nearest 0.0001g using a Sartorius Acculab balance.

SG mass data were analysed using a linear mixed effects model in the R [2] package LME4 [3], with male selection line (long or short), body side (left or right) and the interaction between them as fixed effects. Body mass was included as a covariate. Male identity was included as a random effect to account for two measures of SG mass per male. P values were obtained using the package languageR [4].

There was no difference in SG mass between long and short sperm males (table S4), indicating that the amount of sperm available for copulation is not influenced by selection line. Overall, the right SGs weighed less than the left SGs, but this difference was non significant. The interaction between male selection line and the body side of the SG had no effect on SG mass and was removed from the final model.

Table S4. Results of the linear mixed effects model analysing the effect of male selection line and the body side on seminal glomera mass.

| Model parameter | Mean | HP95^a^ (upper, lower) | pMCMC |
| --- | --- | --- | --- |
| Intercept | 0.0065 | (0.0065, 0.0026) | --- |
| Selection line: short | -0.0009 | (-0.0009 -0.0019) | 0.0706 |
| Body side: right | -0.0004 | (-0.0004, -0.0012) | 0.2790 |
| Body mass (g) | -0.0002 | (-0.0002, -0.0004) | 0.1558 |

The selection history of the male did not affect SG mass. Data comprise SG masses collected from 12 males that were not part of the sperm competition experiment.

^a^Highest posterior density intervals are akin to 95% confidence intervals.

References

1. Birkhead TR, Pellatt EJ & Fletcher F. 1993 Selection and utilization of spermatozoa in the reproductive tract of the female zebra finch *Taeniopygia guttata*. *J. Reprod. Fert.* **99**, 593-600.

2. R Development Core Team. 2012 R: A language and environment for statistical computing. R Foundation for Statistical Computing, Vienna, Austria. See <http://www.R-project.org/>.

3. Bates DM, Maechler M & Bolker BM. 2012 lme4: Linear mixed-effects models using S4 classes. R package version 0.999999-0. See http://CRAN.R- project.org/package=lme4.

4. Baayen R. 2011 languageR: Data sets and functions with "Analyzing Linguistic Data: A practical introduction to statistics". R package version 1.4. http://CRAN.R-project.org/package=languageR.
