# Supplementary material for: Long sperm fertilize more eggs in a bird
Source: Proc Biol Sci. 2015 Jan 22;282(1799):20141897. doi: 10.1098/rspb.2014.1897 (PMC4286041; doi:10.1098/rspb.2014.1897)
Supplement: Quantifying competitive success [file rspb20141897supp3.docx]

Quantifying competitive success: additional methods

a) Long and short sperm embedded on the outer perivitelline layer

Sperm embedded in the outer perivitelline layer (OPVL) of eggs can easily be photographed (Figure S2) using a microscope and camera. By measuring sperm total length (head plus midpiece plus tail), or flagellum length (midpiece plus tail) with image analysis software (e.g. [1]), and comparing these lengths against the known ranges of lengths for a particular male, sperm can be confidently assigned to the long sperm or the short sperm male. We assigned 4420 sperm to one of the two competing males. Some sperm could not be confidently assigned to either male (n = 960) due to debris on the OPVL preventing measurement; we assumed that debris would affect long and short sperm equally (this assumption was validated by the similar pattern of results obtained from the paternity analysis).


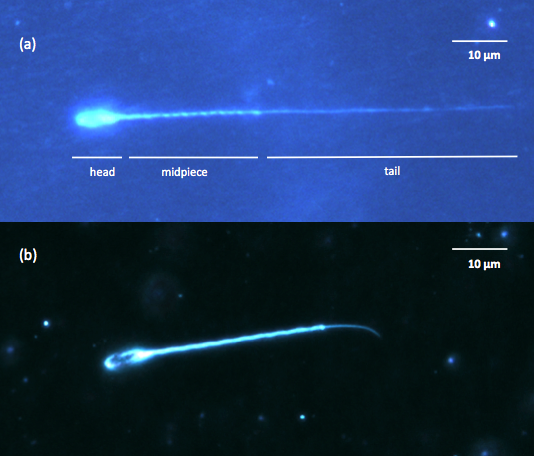


Figure S2. Sperm embedded on the outer perivitelline layer of two different eggs, visualised using a combination of fluorescence and darkfield microscopy at 400x magnification. The scale bar represents 10 μm in both images. (a) a long sperm measuring approximately 73 μm in length. (b) a short sperm measuring approximately 51 μm in length. The head, midpiece and tail can be clearly identified and are indicated by the white bars in (a). Both total length and flagellum (midpiece plus tail) length are easily measured using image analysis software (e.g. [1]). The images above demonstrate that the long and short sperm can also be distinguished visually.

b) Genotyping

DNA was extracted from all embryos, and from all potential parents (using blood samples obtained under licence) using the ammonium acetate extraction protocol [2]. PCR reactions were run with eight microsatellite markers (TG01-124, TG01-147, TG03-002, TG05-053, TG07-022, TG13-009, TG13-017 and Z-002E (note that Z-002E is a sex determining marker and was not used in the parentage analysis) in a pre-optimised multiplex [3]. Markers of similar size were distinguished by coloured fluorolabels: either 6-FAM or HEX (Geneworks). Each 2 μl PCR contained 1 μl of air-dried genomic DNA (20 ng/µl), 1 μl of primer mix containing 0.2 μM of each fluoro-labelled forward and reverse primer, and 1 μl of Quigen master mix (QIAGEN Inc.) [4]. Each well was covered with a drop of mineral oil. DNA was amplified on a DNA Engine Tetrad 2 thermocycler (MJ Research, Bio-Rad, Hemel Hampstead, Herts, UK).

The thermocycling profile was as follows: an initial denaturing incubation (95^o^C for 15 minutes) followed by 44 cycles at the following temperatures: 94^o^C for 30 s, 56^o^C for 1 minute 30 s and 72^o^C for 1 minute 30 s. This was followed by a final extension step at 72^o^C for 10 minutes. The PCR products were diluted to 1 in 800 and 1 µl of this dilution added to 9.5 µl mixture of formamide and ROX 500 size standards (Applied Biosystems, Warrington, UK). The samples were denatured at 95^o^C for 3 minutes then immediately placed in an iced water bath to prevent re-annealing, before being sequenced using an ABI 3730 48-well capillary sequencer (Applied Biosystems, California, USA. The reaction products were visualised and scored for each of the marker loci using GeneMapper v 3.7 ® (Applied Biosystems, California, USA).

c) Paternity assignment

Cervus v 3.0.3 [5] was used to assign paternity to each embryo. An allele frequency analysis was carried out on 313 individuals, using seven microsatellite markers (the sex determining marker Z-002E was not used). Two microsatellite markers (TG05-053 and TG13-009) appeared to segregate for null alleles and were removed from the dataset. The paternity analysis was therefore carried out using the remaining 5 microsatellite markers (TG01-124, TG01-147, TG03-002, TG05-053 and TG07-022). Typing error rates were estimated to be 4% across the 5 loci by examining Mendelian inconsistencies between females and embryos. To be conservative, we assumed a genotyping error rate of 5% during paternity analysis. Overall, the genotyping success rates were 90.2% and the five loci contained between 3 and 5 alleles. The expected heterozygosities ranged from 0.28-0.69. All loci were in Hardy-Weinberg Equilibrium. We excluded any embryos that were typed at two or fewer loci (n = 5).

We assigned paternity to each embryo using trio wise assignments, where the female bird was included as the known mother and the pair of males as the candidate sires. We assumed that the two candidate males were not relatives because they originated from different selection lines that had undergone strict selective breeding for three generations.

Using a likelihood approach, males were assigned paternity to an embryo with at least 80% confidence when a Delta score of zero or higher was achieved (n = 166). These thresholds were lower than are seen in most parentage inference analyses because we only had two candidate males for each offspring (i.e. the task of assigning parentage is easier than in most studies). Delta scores are calculated such that if the second male has a LOD (logarithm of the odds) score that is <0, then Delta is LOD 1^st^ male – 0. An inspection of the LOD scores of both candidate males showed that in most cases the non-assigned male had a negative LOD score, making it very unlikely to be the true parent. This gives an extra degree of confidence in our assignments.

Genotyping mismatches were observed between embryos and the assigned males in twenty-two cases. One mismatch was observed in twenty-one embryos, and two mismatches were recorded in a single embryo. These genotyping errors are accounted for in the likelihood assignment method. Some mismatching is to be expected given the low, but nonzero error rates. If a trio of individuals are typed at 5 loci, which are 15 genotypes in total, assuming a 4% error rate, we expect 54% of trios to contain zero genotyping errors under a binomial distribution. It is inevitable that there are some mismatches between father-mother-offspring trios even when the true father is assigned. We found that the assigned father usually had a positive LOD score and no mismatches, while the non-assigned father had a negative LOD score and one or more mismatches with the offspring.

References

1. Schneider CA, Rasband WS & Eliceiri KW. 2012 NIH Image to ImageJ: 25 years of image analysis. *Nature Methods* **9**, 671-675. (doi:10.1038/nmeth.2089).

2. Bruford MW, Hanotte O, Brookfield JFY & Burke T. 1998 Multilocus and single-locus DNA fingerprinting. In *Molecular Genetic Analysis of Populations: A Practical Approach*. 2nd edition, (ed A. R. Hoelzel), pp. 287-336. IRL Press, Oxford, UK.

3. Dawson DA, Horsburgh GJ, Kupper C, Stewart IRK, Ball AD, Durrant KL, Hansson B, Bacon I, Bird S, Klein A., et al. 2010 New methods to identify conserved microsatellite loci and develop primer sets of high cross-species utility - as demonstrated for birds. *Mol. Ecol. Resour.* **10**, 475-494. (doi:10.1111/j.1755-0998.2009.02775.x).

4. Kenta T, Gratten J, Hinten G, Slate J, Butlin RK & Burke T. 2008 Multiplex SNP-SCALE: a cost-effective medium-throughput SNP genotyping method. *Mol. Ecol. Resour.* **8**, 1230–1238.

5. Kalinowski ST, Taper ML & Marshall TC. 2007 Revising how the computer program CERVUS accommodates genotyping error increases success in paternity assignment. *Mol. Ecol. Notes* **16**, 1099-1006. (doi:10.1111/j.1365-294x.2007.03089.x).
