# Supplementary material for: Long sperm fertilize more eggs in a bird
Source: Proc Biol Sci. 2015 Jan 22;282(1799):20141897. doi: 10.1098/rspb.2014.1897 (PMC4286041; doi:10.1098/rspb.2014.1897)
Supplement: Relatedness between experimental birds [file rspb20141897supp4.docx]

Relatedness of experimental birds

The birds used in this study were part of a long-term study with complete pedigree data going back to the mid-1980s. Therefore, we estimated relatedness of all experimental birds used in this study using the R package Pedantics [1], which has been specifically written for quantitative genetics analysis of pedigree data. We estimated the A matrix (the relationship matrix) for all birds in the pedigree, and then examined relatedness values between (i) males and females within each trio and (ii) between the two competing males in each trio. Mean relatedness levels were low in both cases (mean ± SD): mean relatedness between paired females and males = 0.0371 ± 0.0557: mean relatedness between competing males = 0.0026 ± 0.007.

We then examined the relatedness scores of each group of males and females according to their selection line, e.g. the relatedness of the long sperm male and the long line female, and so on. The mean relatedness scores (mean ± SD) for each group are as follows: (i) long line females and long sperm males: 0.022 ± 0.022, (ii) long line females and short sperm males: 0.023 ± 0.04, (iii) short line females and long sperm males: 0.037 ± 0.006, and (iv) short line females and short sperm males: 0.09 ± 0.07. This information is also summarised in Figure S1.

The mean relatedness scores of the short line females and short sperm males is higher than the other male-female line combinations, specifically, the relatedness scores of 5 out of the 10 female – male pairs were between 0.125 and 0.1875. Although these values are greater than the other three groups of female – male pairings, the values are still small compared relatedness scores of full siblings (approximately 0.5, see figure S1). These slightly higher relatedness scores are extremely unlikely to have biased our results for the following reasons: (i) A recent paper [2] analysed whether marker based paternity assignment favoured assignment towards heterozygous and unrelated males, and found that, although biases can occur, they may be in either direction, i.e. towards the related or the unrelated males.

(ii) Importantly, when biases did occur, they were worse when exclusion methods were used, compared to when likelihood methods were employed – note that in this study we used a likelihood assignment method.

(iii) Additionally, Wang et al. 2010 [1] also found no evidence to suggest that the likelihood approaches would preferentially assign parentage to the unrelated males rather than the related male.

(iv) Previous work from our lab [3] also demonstrated that non-competitive fertilisation success of brother-sister matings, where relatedness between males and females was much higher (approximately 0.5) than is reported in the present study, was equal to the fertilisation success of unrelated pairs. In another bird, the mallard duck *Anas platyrhynchos*, Denk et al. 2005 [4] carried out competitive fertilisation trials where females were inseminated with the sperm from a brother and an unrelated male. The relatedness of the male and female did not affect the fertilisation success of the males.

In summary, in our study, although the short line females tended to be more closely related to the short sperm males, there is no evidence that this would have biased our parentage analysis in favour of assigning parentage to the long sperm males.

Figure S1. The relatedness scores of the four groups of males and females (grouped by selection line) from the sperm competition experiment. The labels across the x axis are the selection line of the female and male, respectively. The higher relatedness of the short line females and short sperm males are significantly less than the relatedness we would expect between full sibling pairings (approximately 0.5 – refer to the maximum limit on the y axis). The horizontal black line across each bar represents the median relatedness value of each group, and the dashed lines show the standard deviation. See main text above for mean values of relatedness in each group.

References

1. Morrissey MB, Wilson AJ. 2010 Pedantics: an r package for pedigree-based genetic simulation and pedigree manipulation, characterization and viewing. *Mol. Ecol. Resours*. **10**(4):711-719. (doi:10.1111/j.1755-0998.2009.02817.x).

2. Wang JL. 2010 Do marker-based paternity assignments favour heterozygous and unrelated males? *Mol. Ecol*. **19**, 1898-1913. (doi:10.1111/j.1365-294X.2010.04601.x).

3. Hemmings NL, Slate J & Birkhead TR. 2012 Inbreeding causes early death in a passerine bird. *Nature Communications* **3**. (doi:86310.1038/ncomms1870).

4. Denk AG, Holzmann A, Peters A, Vermeirssen ELM & Kempenaers B. 2005 Paternity in mallards: effects of sperm quality and female sperm selection for inbreeding avoidance. *Behav. Ecol*. **16**, 825-833. (doi:10.1093/beheco/ari065).
