# Supplementary material for: Long sperm fertilize more eggs in a bird
Source: Proc Biol Sci. 2015 Jan 22;282(1799):20141897. doi: 10.1098/rspb.2014.1897 (PMC4286041; doi:10.1098/rspb.2014.1897)
Supplement: Sperm competition experiment summary tables [file rspb20141897supp5.docx]

Summary data: sperm competition experiment

Summary data from the sperm competition trials are presented below. Table S5 shows the proportions of long sperm reaching the ova, and embryos sired by the long sperm male in each of the four pairing combinations. Table S6 shows the model output of the generalised linear mixed model (GLMM) analysing the effect of male mating order and female selection line on the embryo paternity. Non-significant interactions were removed sequentially using log likelihood tests.

Table S5. The proportion of long sperm observed on the OPVL and the proportion of paternity achieved by the long sperm male across each pairing combination. More long sperm reached the ovum, and consequently fertilised the ovum compared to short sperm in three out of four mating combinations.

| **Female line** | **Copulation order**  **(1^st^ male: 2^nd^ male)** | **Clutches**  **(n_total_ = 30)** | **Proportion sired by**  **long male ± SEM** | |
| --- | --- | --- | --- | --- |
|  |  |  | **OPVL** | **Paternity** |
| Long | Short – Long | 8 | 0.45 ± 0.05 | 0.46 ± 0.17 |
| (n = 8) | Long – Short | 5 | 0.68 ± 0.04 | 0.72 ± 0.17 |
| Short | Short – Long | 8 | 0.66 ± 0.05 | 0.74 ± 0.15 |
| (n = 10) | Long – Short | 9 | 0.59 ± 0.05 | 0.67 ± 0.14 |

Table S6. Results of the GLMM analysing the effect of male mating order and female line on male fertilisation success. Male fertilisation success was determined by an interaction between male mating order (either first or second) and female selection line (long or short).

|  | **Model estimate ± SEM** | **z** | **p** |
| --- | --- | --- | --- |
| Male mating order | 0.57 ± 0.59 | 0.96 | 0.33 |
| Female line | -0.40 ± 1.44 | -0.28 | 0.78 |
| Male mating order x female line | 3.60 ± 1.12 | 3.20 | **0.001** |

Data comprise 166 eggs from 18 females. Reanalysing the data including only females that produced clutches in both mating rounds produced qualitatively similar results (data not shown).
